# Supplementary material for: Demonstration of Co-Infection and Trans-Encapsidation of Viral RNA In Vitro Using Epitope-Tagged Foot-and-Mouth Disease Viruses
Source: Viruses. 2021 Dec 3;13(12):2433. doi: 10.3390/v13122433 (PMC8708420; doi:10.3390/v13122433)
Supplement: Supplementary file 1 [file viruses-13-02433-s001.zip › viruses-1439254-supplementary.pptx]

## Slide 1
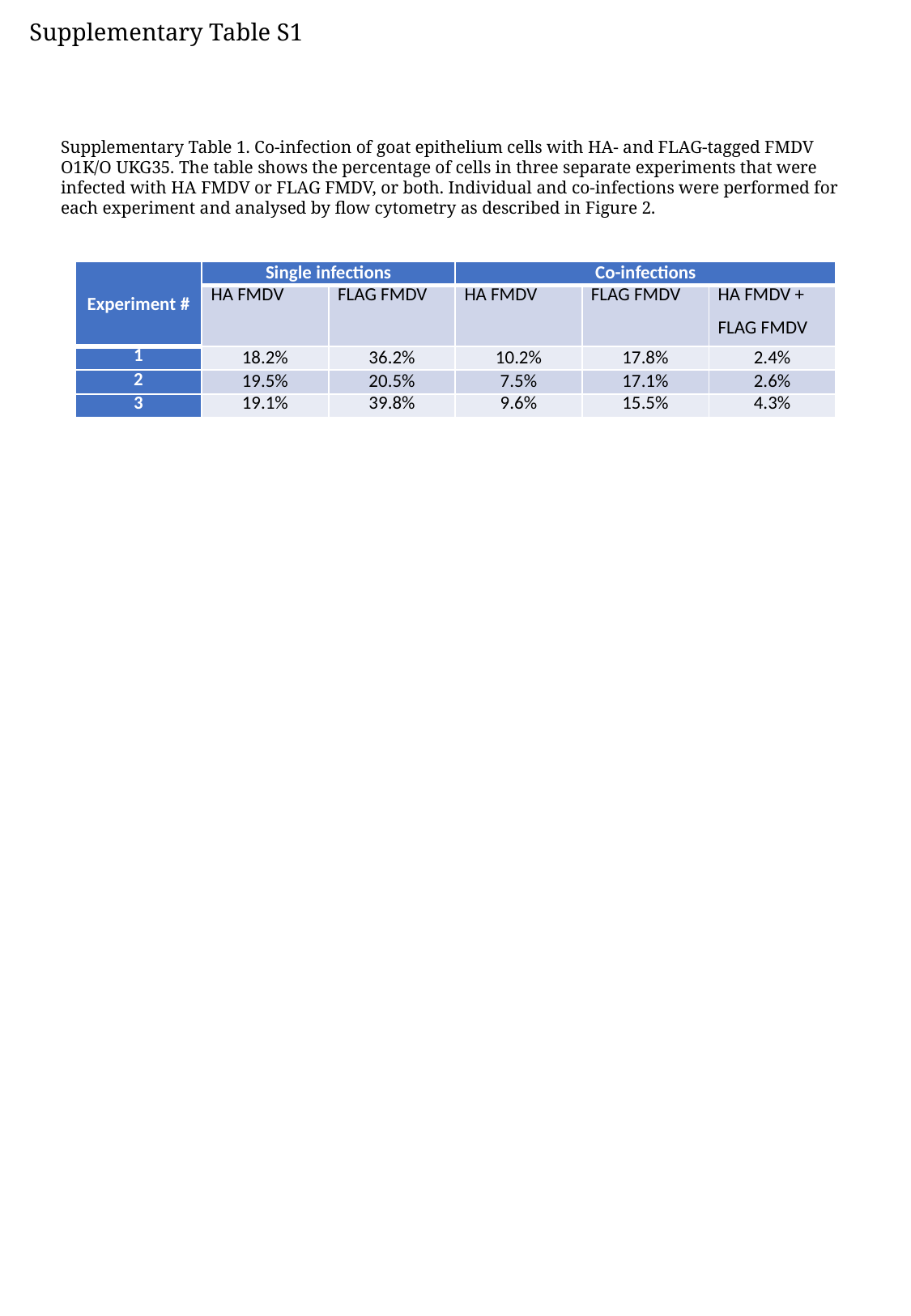

Supplementary Table S1
Supplementary Table 1. Co-infection of goat epithelium cells with HA- and FLAG-tagged FMDV O1K/O UKG35. The table shows the percentage of cells in three separate experiments that were infected with HA FMDV or FLAG FMDV, or both. Individual and co-infections were performed for each experiment and analysed by flow cytometry as described in Figure 2.
| Experiment # | Single infections | | Co-infections | | |
| --- | --- | --- | --- | --- | --- |
| | HA FMDV | FLAG FMDV | HA FMDV | FLAG FMDV | HA FMDV + FLAG FMDV |
| 1 | 18.2% | 36.2% | 10.2% | 17.8% | 2.4% |
| 2 | 19.5% | 20.5% | 7.5% | 17.1% | 2.6% |
| 3 | 19.1% | 39.8% | 9.6% | 15.5% | 4.3% |

## Slide 2
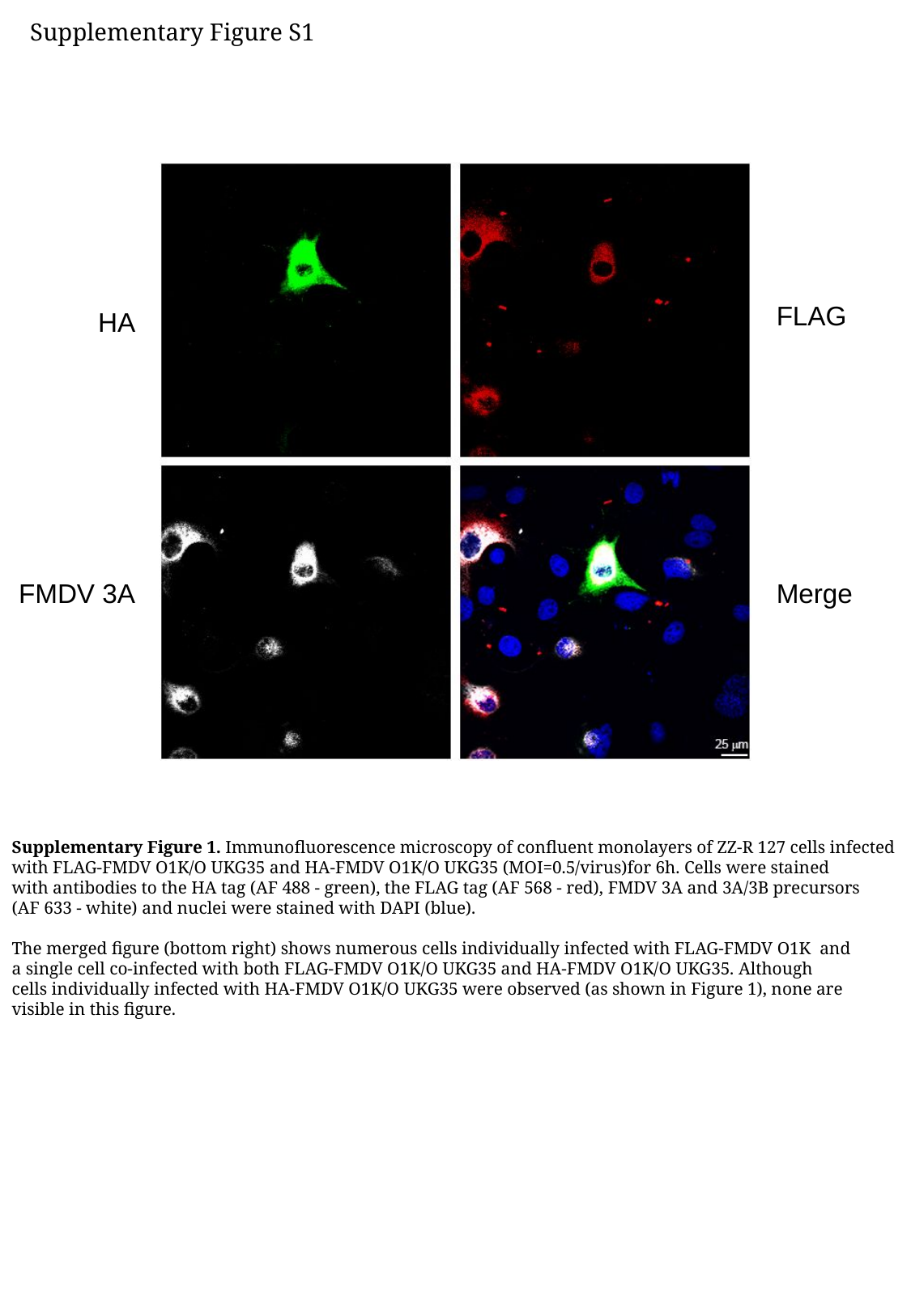

Supplementary Figure S1
FLAG
HA
FMDV 3A
Merge
Supplementary Figure 1. Immunofluorescence microscopy of confluent monolayers of ZZ-R 127 cells infected
with FLAG-FMDV O1K/O UKG35 and HA-FMDV O1K/O UKG35 (MOI=0.5/virus)for 6h. Cells were stained
with antibodies to the HA tag (AF 488 - green), the FLAG tag (AF 568 - red), FMDV 3A and 3A/3B precursors
(AF 633 - white) and nuclei were stained with DAPI (blue).
The merged figure (bottom right) shows numerous cells individually infected with FLAG-FMDV O1K and
a single cell co-infected with both FLAG-FMDV O1K/O UKG35 and HA-FMDV O1K/O UKG35. Although
cells individually infected with HA-FMDV O1K/O UKG35 were observed (as shown in Figure 1), none are
visible in this figure.

## Slide 3
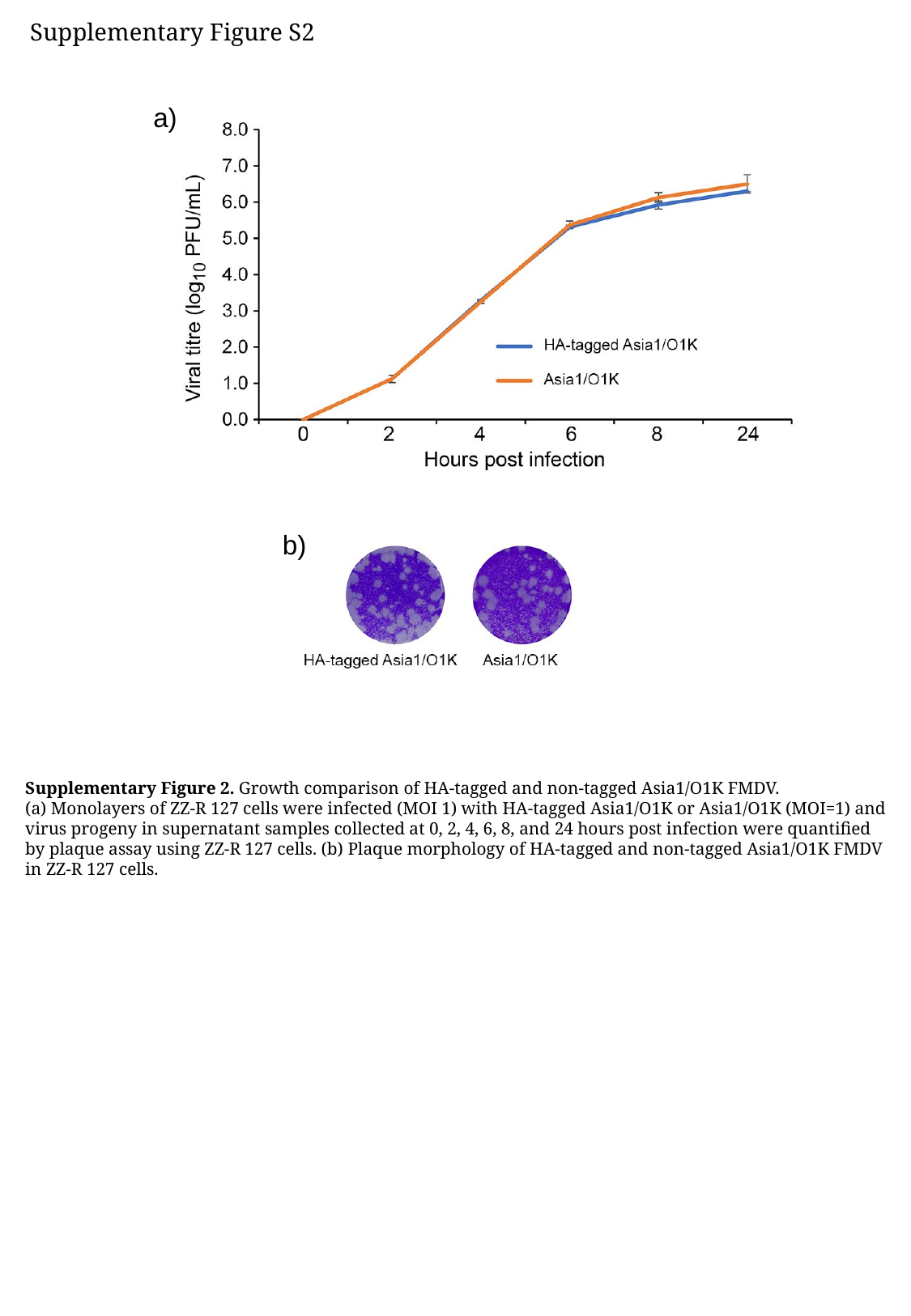

Supplementary Figure S2
a)
b)
Supplementary Figure 2. Growth comparison of HA-tagged and non-tagged Asia1/O1K FMDV.
(a) Monolayers of ZZ-R 127 cells were infected (MOI 1) with HA-tagged Asia1/O1K or Asia1/O1K (MOI=1) and
virus progeny in supernatant samples collected at 0, 2, 4, 6, 8, and 24 hours post infection were quantified
by plaque assay using ZZ-R 127 cells. (b) Plaque morphology of HA-tagged and non-tagged Asia1/O1K FMDV
in ZZ-R 127 cells.
